# PCNA Ubiquitination Is Important, But Not Essential for Translesion DNA Synthesis in Mammalian Cells

**DOI:** 10.1371/journal.pgen.1002262

**Published:** 2011-09-08

**Authors:** Ayal Hendel, Peter H. L. Krijger, Noam Diamant, Zohar Goren, Petra Langerak, Jungmin Kim, Thomas Reißner, Kyoo-young Lee, Nicholas E. Geacintov, Thomas Carell, Kyungjae Myung, Satoshi Tateishi, Alan D'Andrea, Heinz Jacobs, Zvi Livneh

**Affiliations:** 1 Department of Biological Chemistry, Weizmann Institute of Science, Rehovot, Israel; 2 Division of Immunology, The Netherlands Cancer Institute, Amsterdam, The Netherlands; 3 Department of Radiation Oncology and Pediatric Oncology, Dana-Farber Cancer Institute, Harvard Medical School, Boston, Massachusetts, United States of America; 4 Department of Chemistry and Biochemistry, Ludwig Maximilians-University Munich, Munich, Germany; 5 Genome Instability Section, Genetics and Molecular Biology Branch, National Human Genome Research Institute, National Institutes of Health, Bethesda, Maryland, United States of America; 6 Chemistry Department, New York University, New York, United States of America; 7 Institute of Molecular Embryology and Genetics, Kumamoto University, Kumamoto, Japan; University of Washington, United States of America

## Abstract

Translesion DNA synthesis (TLS) is a DNA damage tolerance mechanism in which specialized low-fidelity DNA polymerases bypass replication-blocking lesions, and it is usually associated with mutagenesis. In *Saccharomyces cerevisiae* a key event in TLS is the monoubiquitination of PCNA, which enables recruitment of the specialized polymerases to the damaged site through their ubiquitin-binding domain. In mammals, however, there is a debate on the requirement for ubiquitinated PCNA (PCNA-Ub) in TLS. We show that UV-induced Rpa foci, indicative of single-stranded DNA (ssDNA) regions caused by UV, accumulate faster and disappear more slowly in *Pcna^K164R/K164R^* cells, which are resistant to PCNA ubiquitination, compared to *Pcna^+/+^* cells, consistent with a TLS defect. Direct analysis of TLS in these cells, using gapped plasmids with site-specific lesions, showed that TLS is strongly reduced across UV lesions and the cisplatin-induced intrastrand GG crosslink. A similar effect was obtained in cells lacking Rad18, the E3 ubiquitin ligase which monoubiquitinates PCNA. Consistently, cells lacking Usp1, the enzyme that de-ubiquitinates PCNA exhibited increased TLS across a UV lesion and the cisplatin adduct. In contrast, cells lacking the Rad5-homologs Shprh and Hltf, which polyubiquitinate PCNA, exhibited normal TLS. Knocking down the expression of the TLS genes *Rev3L*, *PolH*, or *Rev1* in *Pcna^K164R/K164R^* mouse embryo fibroblasts caused each an increased sensitivity to UV radiation, indicating the existence of TLS pathways that are independent of PCNA-Ub. Taken together these results indicate that PCNA-Ub is required for maximal TLS. However, TLS polymerases can be recruited to damaged DNA also in the absence of PCNA-Ub, and perform TLS, albeit at a significantly lower efficiency and altered mutagenic specificity.

## Author Summary

DNA damage can block replication and lead to mutations, genomic instability, and cancer. In cases when the removal of DNA damage and restoration of the original sequence prior to replication is impossible, cells utilize DNA damage tolerance mechanisms, which help replication to bypass the lesions. A major universal tolerance mechanism is translesion DNA synthesis (TLS), in which specialized low-fidelity DNA polymerases elongate the DNA across the lesion. This is a double-edged sword because the price of completing replication is an increased risk of point mutations opposite the lesion. Thus, TLS regulation is critical for preventing an escalation in mutation rates. A key element in TLS regulation is the attachment of a small protein called ubiquitin to the PCNA protein, a sliding DNA clamp that tethers the DNA polymerases to DNA, which functions to recruit the TLS DNA polymerase to the damaged site in DNA. While in yeast this modification of PCNA is crucial for TLS, there is a debate about its importance in mammals. Here we show that in mammalian cells the modification of PCNA by ubiquitin is important, but there exist secondary yet significant TLS mechanisms that operate in its absence and have an altered mutational outcome.

## Introduction

Translesion DNA synthesis is a universal DNA damage tolerance mechanism, which enables continuous functioning of replication despite the presence of DNA lesions. While the replisome might be able to bypass lesions that cause minor changes in the structure of DNA, lesions which are bulky or cause significant DNA deformation, block replication. Such lesions are bypassed by specialized low-fidelity DNA polymerases, which are capable of replicating across DNA damage due to a flexible structure and promiscuous active site that allows lesion bypass at the cost of increased mutagenesis. At least 5 specialized DNA polymerases are involved in TLS in mammalian cells, namely DNA polymerases η, κ, ι, ζ and REV1, however, the number may be as high as 10. Each polymerase exhibits a certain range of specificity towards various types of DNA lesions, with some overlap [1]–[4]. TLS typically operates in two-polymerase reactions, in which the first polymerase inserts a nucleotide opposite the lesion, and the second polymerase, usually DNA polymerase ζ (polζ), extends beyond the lesion [5]–[7]. The biological importance of TLS is indicated by the essentiality of polζ for mouse development [8], and the high cancer predisposition caused by germ-line mutations in the *POLH* gene (encoding DNA polymerase η; polη) in humans [9], [10]. TLS must be tightly regulated to prevent an escalation in mutation rates. Although TLS regulation is not fully understood, it does appear to be regulated primarily at the posttranslational level, and involves the ubiquitination of PCNA, the sliding DNA clamp that tethers DNA polymerases to the DNA [11]–[14]. In addition, TLS is regulated by the p53 tumor suppressor, which exerts its effect primarily via its target p21 protein [15]. The latter is a cell cycle inhibitor, which exerts its regulatory effect on TLS via its interactions with PCNA [15], and cyclin-dependent kinases [16]. Together p53 and p21 restrain the extent of TLS, but make it more accurate, thereby reducing the mutagenic load of TLS [15].

**Figure 1:**
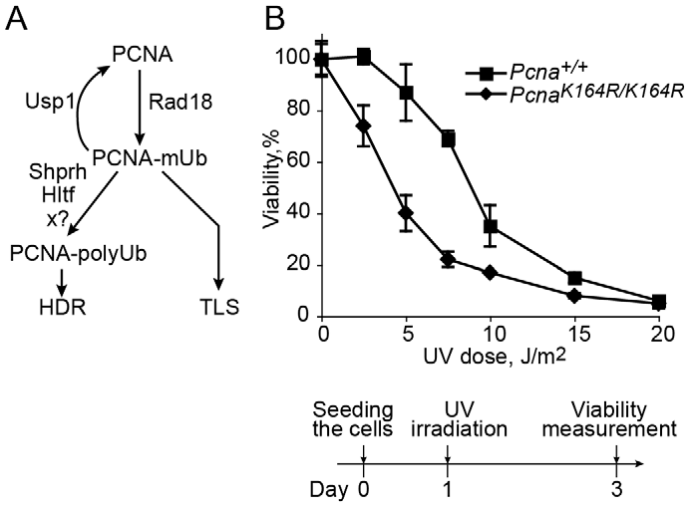
UV sensitivity of mouse embryo fibroblasts carrying the *Pcna^K164R/K164R^* mutation. (A) Outline of ubiquitination and deubiquitination reactions of PCNA. Treatment with DNA-damaging agents, such as UV light, induces monoubiquitination of PCNA at Lys164 by the Rad18 E3 ligase. A common model suggests that the monoubiquitinated PCNA (PCNA-mUb) directly recruits TLS polymerases enabling translesion DNA synthesis. The deubiquitinating enzyme Usp1 removes the ubiquitin from PCNA-mUb, thereby negatively regulating the level of PCNA-Ub. The Rad5 homologs Shprh and Hltf, and an unidentified additional E3 ligase (marked x?) can extend the ubiquitin chain of PCNA-mUb, and the polyubiquitinated PCNA (PCNA-polyUb) thus formed promotes homology-dependent (template switch) error-free damage tolerance. (B) *Pcna^+/+^* and *Pcna^K164R/K164R^* MEFs were irradiated at the indicated UV doses, and assayed for viability after 48 hours by measuring the level of cellular ATP. Each point represents the average of 3 independent experiments.

A key regulatory element in TLS is the monoubiquitination of PCNA at lysine 164 in response to treatment with DNA damaging agents. In *S. cerevisiae* this reaction is carried out by the Rad6-Rad18 E2-E3 ubiquitinating enzymes (Figure 1A; [17], [18]), and is critical for the activity of TLS, functioning to recruit TLS polymerases through their ubiquitin-binding domain, and thereby switching from replicative to TLS polymerases [11], [12]. In higher organisms the involvement of ubiquitinated PCNA (PCNA-Ub) in TLS is less clear. Analysis of replication of damaged DNA in chicken DT40 cells suggested that PCNA ubiquitination is involved in filling in of post-replication gaps [19]. However when measured using a plasmid system in DT40 cells carrying the *Pcna^K164R/K164R^* mutation which prevent ubiquitination of PCNA, TLS was normal across a TT 6-4 photoproduct (TT 6-4 PP), a common UV DNA lesion [20]. As for mammals, a common model suggests that similar to *S. cerevisiae*, PCNA-Ub recruits TLS polymerases to the site of DNA damage mediated via their ubiquitin-binding domain [13], [14], [21]–[23]. This model was challenged by studies reporting that mutations in the ubiquitin-binding domain of polη had no effect on its activities, and it is the direct binding of polη to PCNA which is critically important for its activities [24], [25]. In response it was argued that these results can be explained by an effect of artificial overproduction of the mutant polymerase, which suppressed its lower binding affinity [26]. However, later studies reported that complete deletion of the UBZ ubiquitin-binding domain from polη had no effect on its activities, including TLS across a site specific TT cyclobutane pyrimidine dimer (CPD) in a replicative plasmid assay system [27], leaving the controversy unsettled.

In an attempt to resolve the controversy and clarify the role of PCNA-Ub in TLS in mammalian cells we used several assays with mouse embryonic fibroblasts in which specific TLS genes associated with PCNA ubiquitination were manipulated. The experiments reported here show that the main TLS pathway requires PCNA-Ub. However, there exists a secondary but significant TLS pathway, which occurs in the absence of PCNA-Ub, with lower efficiency and altered mutagenic specificity.

## Results

### UV-induced Rpa foci, indicative of replication gaps, accumulate faster and disappear more slowly in mouse embryo fibroblasts carrying the *Pcna^K164R/K164R^* mutation

A *Pcna* mutant in which Lys164 was replaced by an Arg cannot undergo ubiquitination or sumoylation, and was successfully used to study the role of PCNA ubiquitination in TLS in yeast [17], [18] and chicken DT40 cells [19], [28]. The generation of *Pcna^K164R/K164R^* mice [29] provides a similarly effective tool for studying the role of PCNA ubiquitination in TLS in mammalian cells. We first examined the UV sensitivity of *Pcna^K164R/K164R^* mouse embryo fibroblasts (MEF). As can be seen in Figure 1B, the mutant cells were more sensitive than the *Pcna^+/+^* MEF. This suggests that ubiquitination of PCNA at Lys164 is involved in conferring UV resistance in mammalian cells, although it is possible that the effect was caused not only by the lack of ubiquitin, but also by the mutant form of the PCNA.

**Figure 2:**
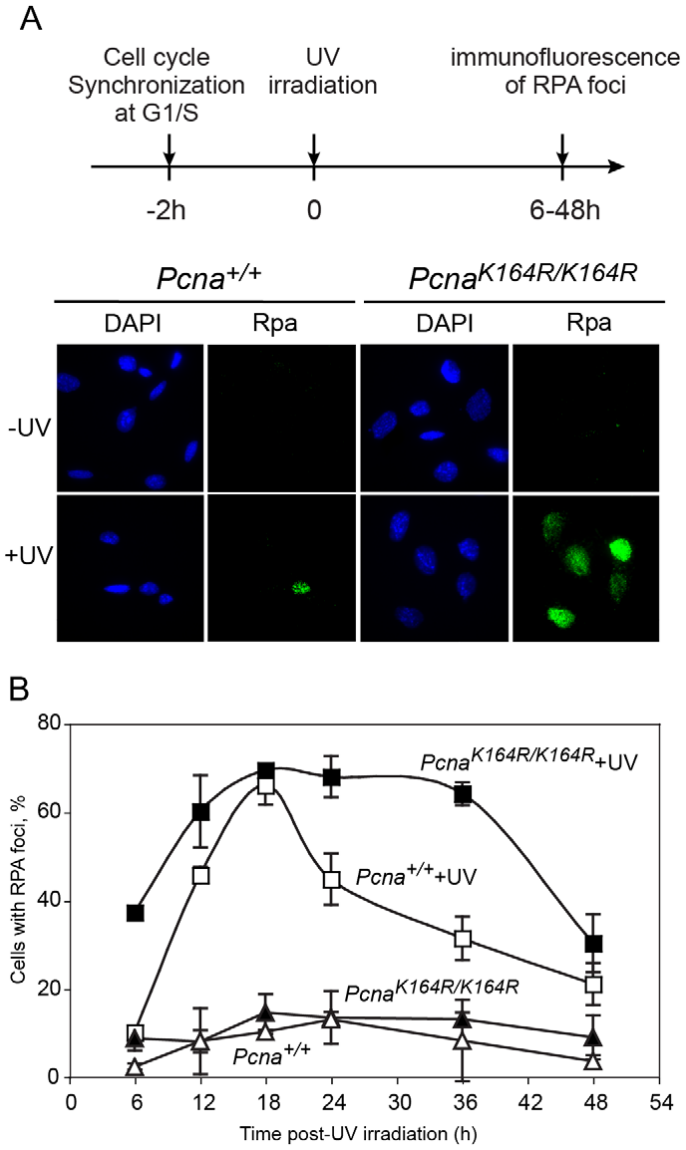
Time course of accumulation and clearance of UV-induced Rpa foci. *Pcna^+/+^* and *Pcna^K164R/K164R^* MEFs were collected at the G1/S boundary by centrifugal elutriation, and allowed to attach to microscope slides. Two hours later they were UV irradiated at a dose of 8 J/m^2^. At the indicated time points cells were pre-extracted and then fixed, immuno-stained with anti-Rpa antibody and DAPI-stained. (A) Representative images of cells stained with DAPI or antibodies against Rpa, either with or without UV irradiation. The timeline of the experiment is shown at the top. (B) Quantification of the percentage of cells with Rpa foci. For each cell line at each time point at least 100 cells were counted and the percentage of cells with Rpa foci was determined. The results are the average of two independent experiments. Error bars represent standard deviation. Full symbols, *Pcna^K164R/K164R^* MEFs; Empty symbols, wild-type MEFs; Squares, UV irradiated cells; Triangles, unirradiated cells.

UV irradiation causes stalling of replication forks and the generation of ssDNA regions in DNA, which may subsequently be broken, thereby forming double strand breaks (DSB). The latter can facilitate a variety of chromosomal rearrangements, causing genomic instability, cancer and cell death. To minimize the formation of DSB, cells employ two major types of DNA damage tolerance mechanisms, namely TLS and HDR (homology-dependent repair, also termed HRR, homologous recombination repair; reviewed in [4], [30]). Of the two, TLS was reported to be the major mechanism for overcoming UV lesions in MEF [31]. Thus, analysis of UV-induced ssDNA regions during replication can be used as a measure for DNA damage tolerance in general, and TLS in particular. To examine the effect of PCNA ubiquitination on DNA damage tolerance we analyzed the formation and clearance of UV-induced ssDNA regions in *Pcna^K164R/K164R^* MEFs compared to *Pcna^+/+^* MEFs. This was done using immunofluorescence staining of endogenous Rpa2, a subunit of the Rpa ssDNA-binding protein, which is a key protein in DNA replication and repair [32], using a protocol previously used in our lab [33]. To focus on gaps formed during replication we isolated by centrifugal elutriation MEFs at the G1/S boundary, UV irradiated them, and let them grow in culture. At various time points after irradiation the cells were harvested, washed to remove unbound Rpa, and then fixed and stained for chromatin-bound Rpa using immunofluorescence (Figure 2A). In unirradiated cells a low background level of Rpa foci was observed (less than 10%; Figure 2B). Presumably the transient nature and short patch of Rpa-bound ssDNA at normal replication forks does not allow detection under our assay conditions. Upon UV irradiation Rpa foci were induced in the two cell types, but at different rates. Thus, by 6 hours post-irradiation nearly 40% of the *Pcna^K164R/K164R^* cells contained Rpa foci, whereas *Pcna^+/+^* cells contained only the background level of 10% Rpa foci (Figure 2B). The observed fraction of cells with Rpa foci at any given time represents the sum of the rates of their formation and disappearance. Therefore, the higher percentage of Rpa foci at early times in *Pcna^K164R/K164R^* cells represents, most likely the sum of a similar rate of formation but a slower rate of disappearance compared to *Pcna^+/+^* cells. The extent of cells with Rpa foci increased for both cell types, reaching its maximum at 18 hours post-irradiation, after which the number of foci declined, indicating a net conversion of the ssDNA regions to double stranded DNA (dsDNA). The disappearance of Rpa foci was significantly slower in the *Pcna^K164R/K164R^* compared to *Pcna^+/+^* MEFs (Figure 2B). Thus, Rpa foci accumulate faster in *Pcna^K164R/K164R^* compared to *Pcna^+/+^* MEFs following UV irradiation, and disappear slower, consistent with a defect in DNA damage tolerance by TLS.

### TLS is reduced in cells carrying the *Pcna^K164R/K164R^* mutant that is resistant to ubiquitination, and it exhibits altered mutagenic specificity

To directly examine the effect of PCNA-Ub on TLS we used a model assay system based on plasmids carrying a gap opposite a defined site-specific DNA lesion. Briefly, cultured cells were transfected with a mixture containing a gapped plasmid with a site-specific lesion in the ssDNA region, a normalizing control plasmid with a gap, but no lesion, and a carrier plasmid (Figure S1). After allowing time for TLS in the mammalian cells, the plasmid content was extracted under alkaline conditions, and after renaturation it was used to transform an indicator *E. coli recA* (TLS-defective) strain. Under these conditions only plasmids that had been fully filled in and ligated in the mammalian cells were able to efficiently transform the bacterial strain. *E. coli* transformants were selected on LB plates containing kanamycin, to select for descendents of the gap-lesion plasmid (kan^R^), and LB containing chloramphenicol, to select for descendents of the normalizing gapped plasmid (cm^R^). The ratio of kan^R^/cm^R^ colonies provided a measure of the efficiency of gap filling by TLS. Colonies were then picked, their plasmid content extracted, and subjected to DNA sequence analysis at the region of the lesion, to determine any sequence changes that had arisen during TLS. This model assay system proved to be very effective in monitoring TLS events, and shares many of the features of chromosomal TLS, including dependence on specific DNA polymerases and regulatory elements of TLS [6], [15], [34]–[36].

**Figure 3:**
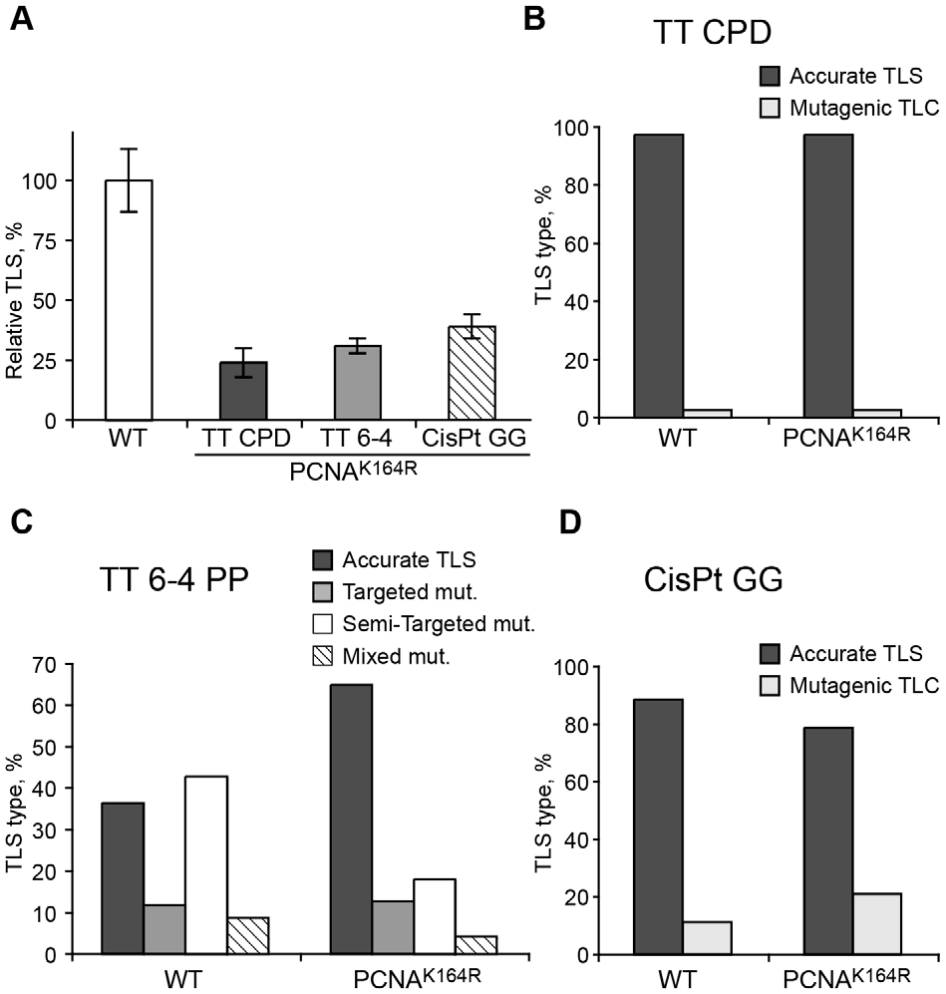
TLS in *Pcna^K164R/K164R^* mouse embryo fibroblasts. (A) MEFs were assayed for TLS as described under Materials and Methods, using the indicated site-specific lesions. TLS extents were given as percentage relative to TLS assayed with isogenic wild type MEFs. Average results of at least three independent experiments are presented. Error bars represent standard deviations. The detailed data are presented in Table S1. (B) Percentage of accurate and mutagenic TLS across a TT CPD in *Pcna^+/+^* and *Pcna^K164R/K164R^* MEFs. (C) Percentage of accurate and mutagenic TLS across TT 6-4 PP. The mutation types shown are: targeted (opposite the lesion), semi-targeted (at the nucleotides flanking the lesion), and mixed (both targeted and semi-targeted). (D) Percentage of accurate and mutagenic TLS across a cisPt-GG adduct. The percentage of events is calculated out of all TLS events. The detailed mutational spectra are presented in Table S2.

Using a gapped plasmid carrying a site-specific TT CPD in the ssDNA region we assayed TLS in *Pcna^K164R/K164R^* MEFs. As can be seen in Figure 3A and Table S1, TLS was reduced 4.4-fold in the mutant *Pcna^K164R/K164R^* cells compared to *Pcna*-proficient MEFs. DNA sequence analysis revealed that 98% of the TLS events in both the mutant and wild-type MEFs were accurate, leading to the insertion of AA opposite the TT CPD, and consistent with the activity of polη (Figure 3B and Table S2). We used the same assay for two additional lesions: a TT 6-4 PP, representing the second most abundant UV lesion, and an intra-strand GG adduct formed by the drug cisplatin (GG-cisPt). TLS across cisPt-GG occurs primarily via a two-polymerase reaction with polη performing insertion opposite the lesion, and polζ performing the extension past the lesion, whereas efficient bypass of TT 6-4 PP does not require polη, but does requires polζ [6]. As can be seen in Figure 3A and Table S1, TLS across a TT 6-4 PP was reduced 3.3-fold in PCNA-Ub deficient MEFs compared to wild-type *Pcna^+/+^* MEFs. DNA sequence analysis revealed that TLS across the TT 6-4 PP in *Pcna^K164R/K164R^* cells was more accurate than in *Pcna^+/+^* cells (64% vs. 35% errors, *P* = 0.0001, χ^2^ test; Figure 3C, Table S2). Analysis of TLS across cisPt-GG revealed that TLS was reduced 2.6-fold in PCNA-Ub deficient MEF compared to wild-type *Pcna^+/+^* MEFs (Figure 3A and Table S1). TLS was largely accurate in both cell types, however, error frequency was twofold higher in the *Pcna^K164R/K164R^* mutant compared to *Pcna^+/+^* MEFs (25% vs 12% errors; *P* = 0.02, χ^2^ test; Figure 3D, Table S2).

### TLS is reduced in MEFs lacking Rad18, but not in MEFs lacking the Rad5 homologs Shprh and Hltf

**Figure 4:**
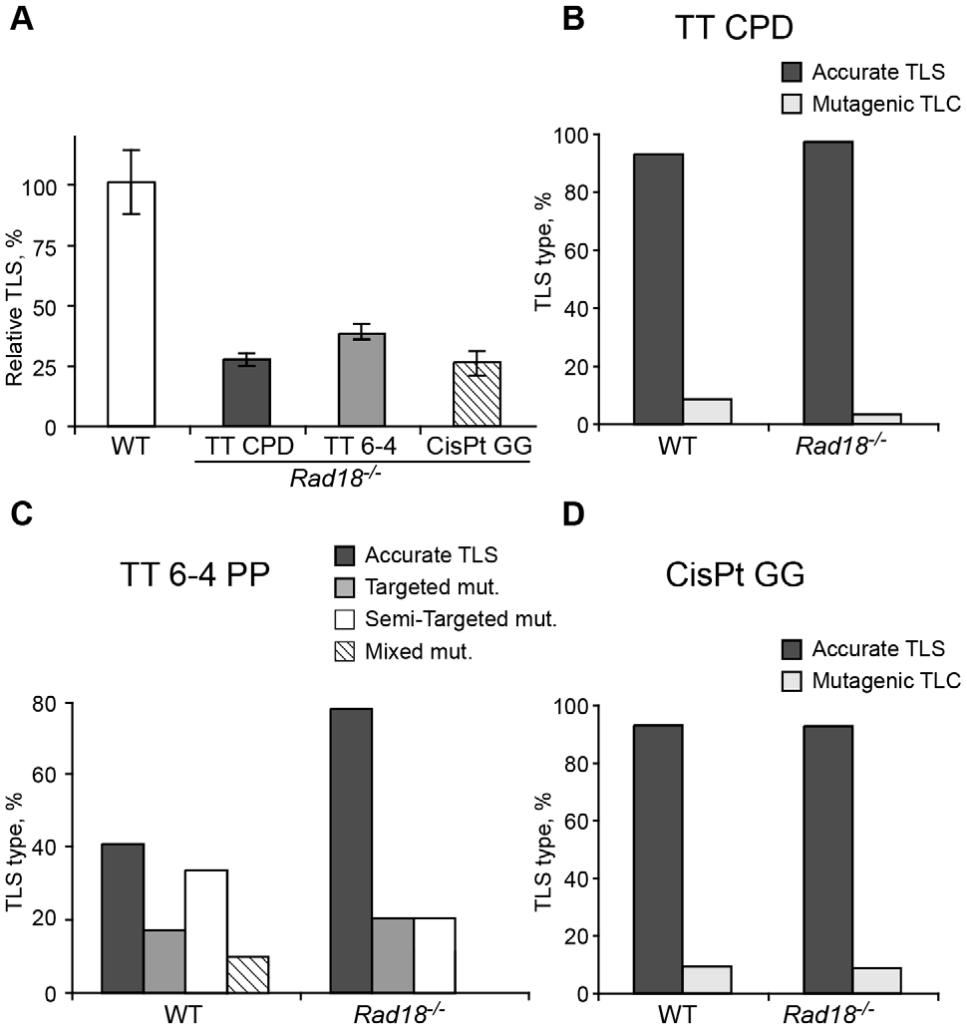
TLS in *Rad8^−/−^* mouse embryo fibroblasts. (A) MEFs were assayed for TLS as described in the legend to Figure 3. The detailed data are presented in Table S3. (B) Percentage of accurate and mutagenic TLS across a TT CPD in *Rad18^+/+^* and *Rad18^−/−^* MEFs. (C) Percentage of accurate and mutagenic TLS across TT 6-4 PP. The mutation types shown are: targeted (opposite the lesion), semi-targeted (at the nucleotides flanking the lesion), and mixed (both targeted and semi-targeted). (D) Percentage of accurate and mutagenic TLS across a cisPt-GG adduct. The percentage of events is calculated out of all TLS events. The detailed mutational spectra are presented in Table S4.

**Figure 5:**
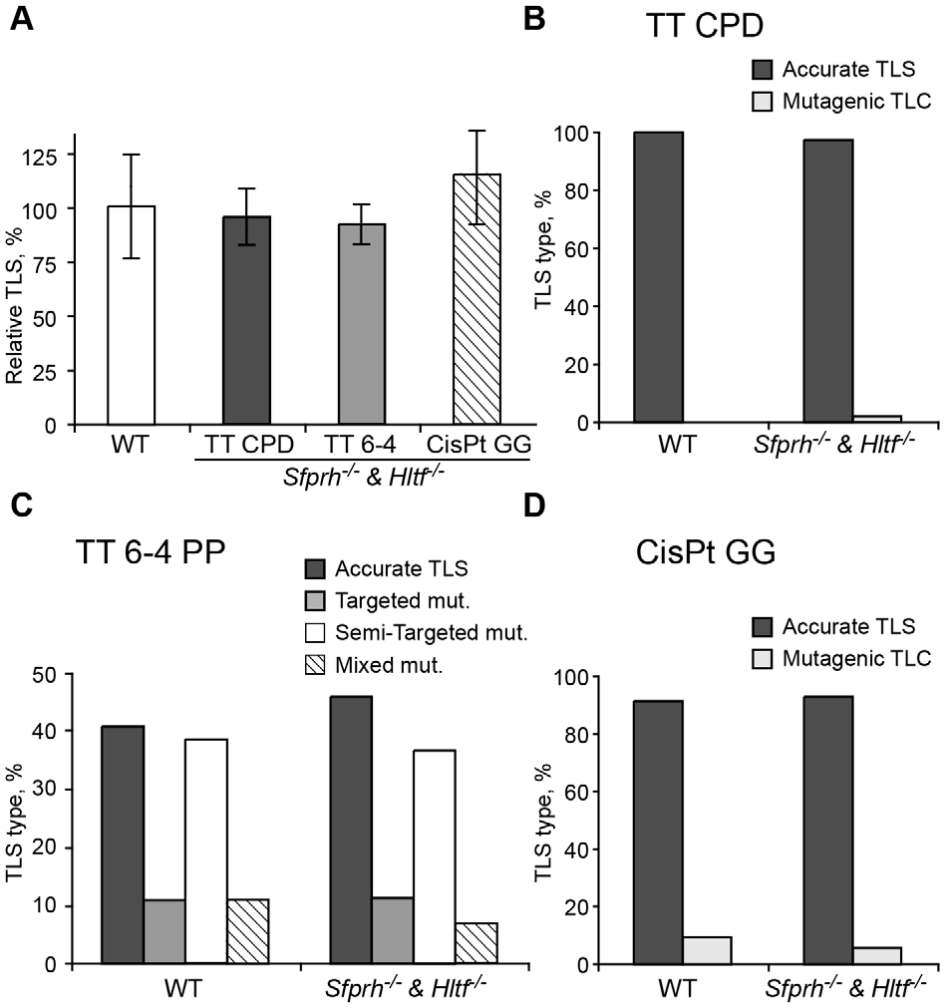
TLS in *Shprh^−/−^Hltf^−/−^* mouse embryo fibroblasts. (A) MEFs were assayed for TLS as described in the legend to Figure 3. The detailed data are presented in Table S5. (B) Percentage of accurate and mutagenic TLS across a TT CPD in *Shprh^+/+^Hltf^+/+^* and *Shprh^−/−^Hltf^−/−^* MEFs. (C) Percentage of accurate and mutagenic TLS across TT 6-4 PP. The mutation types shown are: targeted (opposite the lesion), semi-targeted (at the nucleotides flanking the lesion), and mixed (both targeted and semi-targeted). (D) Frequency of accurate and mutagenic TLS across a cisPt-GG adduct. The percentage of events is calculated out of all TLS events. The detailed mutational spectra are presented in Table S6.

The *Pcna^K164R/K164R^* mutation renders PCNA resistant not only to monoubiquitination, but also to polyubiquitination (Figure 1A) and sumoylation (although PCNA sumoylation was not yet found in mammals). To further analyze the involvement of PCNA modification in TLS we analyzed two additional mutant MEFs: A *Rad18* knockout strain, which lacks the Rad18 E3 ubiquitin ligase that monoubiquitinates PCNA at K164 [37], and an *Shprh^−/−^ Hltf^−/−^* double knockout MEF [38], lacking the two Rad5-homologs, which polyubiquitinate PCNA at K164. As can be seen in Figure 4A and Table S3, TLS in *Rad18^−/−^* MEFs was significantly reduced compared to *Rad18^+/+^* MEFs for each of the three lesions. DNA sequence analysis revealed that mutagenicity of TLS in MEF lacking Rad18 was similar or lower compared to MEF with Rad18 (Figure 4B–4D and Table S4). In contrast, in cells lacking *Shprh* and *Hltf*, the extent of TLS across each of the three lesions was normal (Figure 5A and Table S5), and the mutagenic spectra were similar (Figure 5B–5D; Table S6). These results suggest that maximal TLS indeed requires ubiquitination of PCNA.

### TLS is increased in cells deficient in the Usp1 deubiquitinating enzyme

**Figure 6:**
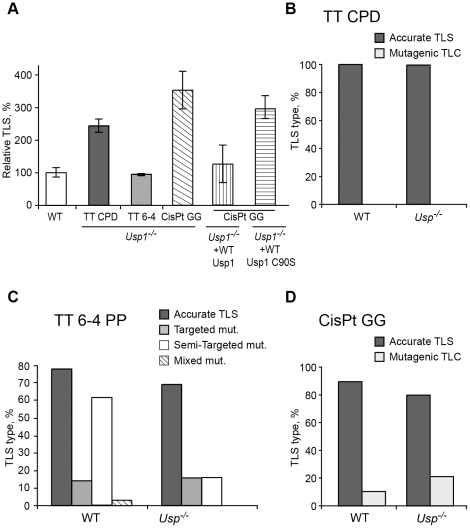
TLS in *Usp1^−/−^* mouse embryo fibroblasts. (A) MEFs were assayed for TLS as described in the legend to Figure 3. The detailed data are presented in Table S7. (B) Percentage of accurate and mutagenic TLS across a TT CPD in *Usp1^+/+^* and *Usp1^−/−^* MEFs. (C) Percentage of accurate and mutagenic TLS across TT 6-4 PP. The mutation types shown are: targeted (opposite the lesion), semi-targeted (at the nucleotides flanking the lesion), and mixed (both targeted and semi-targeted). (D) Frequency of accurate and mutagenic TLS across a cisPt-GG adduct. The percentage of events is calculated out of all TLS events. The detailed mutational spectra are presented in Table S8.

The Usp1 deubiquitinating enzyme was shown to deubiquitinate monoubiquitinated PCNA (PCNA-mUb; Figure 1) [39]. To examine the effect of Usp1 on TLS we assayed TLS in *Usp1^−/−^* MEFs. As can be seen in Figure 6A and Table S7, TLS across a TT CPD was 2.3-fold higher in *Usp1^−/−^* MEFs compared to wild-type MEF. Similarly, TLS across a cisPt-GG adduct was 3.8-fold higher in *Usp1^−/−^* MEFs compared to wild-type MEFs. Interestingly, there was no effect on TLS across the TT 6-4 PP. Complementing the *Usp1^−/−^* MEFs with stably expressed wild-type Usp1 suppressed TLS across cisPt-GG back to wild-type levels, whereas expressing a Usp1 C90S mutant [40] failed to suppress TLS, indicating that the observed effects are indeed due to Usp1 (Figure 6A and Table S7). DNA sequence analysis revealed somewhat different mutagenicity, however with no statistical significance (Figure 6B–6F; Table S8). Thus, the absence or inactivation of the enzyme that deubiquitinates PCNA-mUb caused an *increase* in TLS in 2 out of the 3 lesions studied, in contrast to the *decrease* in TLS caused by the inability to ubiquitinate PCNA. These results are consistent with previous reports that mutations in a UV-irradiated plasmid transfected into mammalian cells were increased when Usp1 was reduced or absent [39], [41].

### PCNA-Ub–independent TLS contributes to UV survival

**Figure 7:**
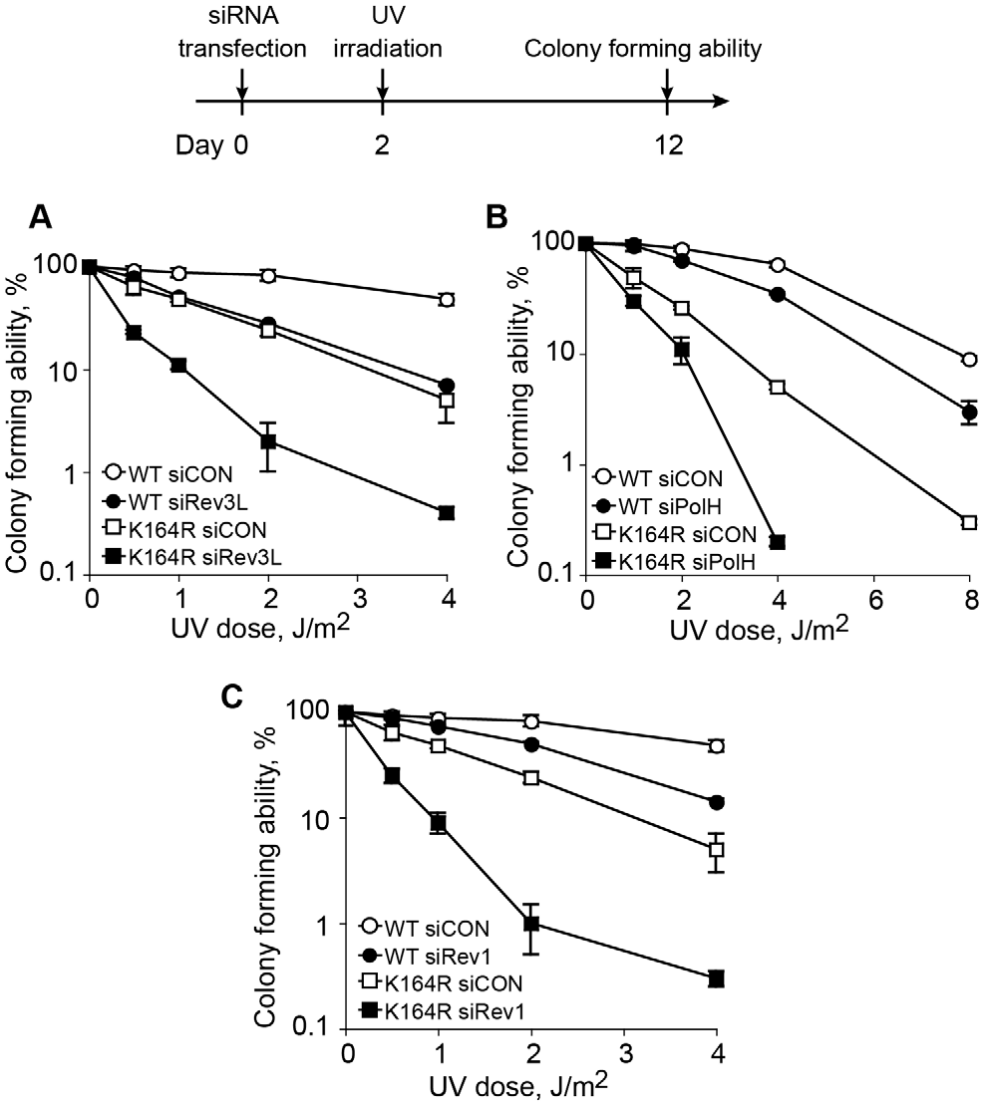
Epistasis analysis of the contributions of ubiquitinated PCNA and TLS DNA polymerases to UV sensitivity. *Pcna^+/+^* and *Pcna^K164R/K164R^* MEFs were transfected with siRNA against the TLS genes *Rev3L* (the catalytic subunit of polζ; A) *PolH* (encoding polη; B) or *Rev1* (C), and after 48 h they were UV irradiated at the indicated doses. Sensitivity was determined 10 days after UV irradiation by measuring colony forming ability. Each point represents the mean of three independent experiments.

The data presented above indicate that although PCNA-Ub is required for maximal TLS in mammalian cells, a significant level of TLS was observed in the absence of PCNA-Ub, suggesting the existence of a PCNA-Ub-independent pathway. We further probed this possibility by assaying UV sensitivity of *Pcna^K164R/K164R^* MEFs in which the expression of specific TLS proteins was knocked-down, using as an assay the ability to form colonies following UV irradiation (Figure 7). As can be seen in Figure 7A–7C, *Pcna^K164R/K164R^* MEFs were more UV sensitive than wild-type MEFs when treated with the control siRNA, consistent with the role of PCNA-Ub in TLS across UV lesions, and with the results presented in Figure 1B. Knocking down the expression of *Rev3L*, encoding the catalytic subunit of polζ, in wild-type MEF caused an increased UV sensitivity (Figure 7A), consistent with previous results [36], and reaching a sensitivity level similar to *Pcna^K164R/K164R^* MEFs treated with a control siRNA. When *Pcna^K164R/K164R^* MEFs were treated with a *Rev3L*-specific siRNA, UV sensitivity further increased (Figure 7A), suggesting the existence of a PCNA-Ub-independent polζ-dependent pathway of TLS. Similar results were obtained when the expression of polη (Figure 7B), or of *Rev1*, an important regulatory protein and a dCMP transferase (Figure 7C), were each knocked down in *Pcna^K164R/K164R^* MEFs. Taken together these results suggest the existence of PCNA-Ub-independent pathways of TLS, which are Polη, Rev1 and/or Polζ dependent.

## Discussion

The debate about the role of PCNA-Ub in polη-promoted TLS in mammalian cells prompted us to address this issue using several mutant mouse cell lines, and several assays. The latter included (1) a TLS assay based on gapped plasmids carrying defined and site-specific lesions; (2) immuno-staining of Rpa foci following UV irradiation of cells at the G1/S boundary of the cell cycle, which assays ssDNA gaps caused by UV lesions; and (3) UV sensitivity as manifested by the ability of irradiated cells to form colonies. Overall we studied three types of lesions, two of which are formed by UV radiation and one by the drug cisplatin, representing three different TLS sub-pathways [6], [7].

The effects of the knockout mutations in each of the mutants analyzed, *Pcna^K164R/K164R^*, *Rad18^−/−^*, *Shprh^−/−^Hltf^−/−^*, and *Usp1^−/−^*, can be attributed to more than one pathway. Thus, the *Pcna^K164R/K164R^* mutant is deficient not only in monoubiquitination, but also in polyubiquitination and sumoylation [42]; The *Rad18^−/−^*, which is deficient in monoubiquitination of PCNA, is known to be deficient in the ubiquitination of other proteins as well (e.g., [43]), and similarly the other mutants may affect several activities. However, the similar effects on TLS of the *Pcna^K164R/K164R^* and *Rad18^−/−^* cells, suggest that ubiquitination rather than sumoylation is involved. Noteworthy, no alternative PCNA ubiquitination site was observed in mouse PCNA [29]. What about the discrimination between monoubiquitination and polyubiquitination of PCNA? The Hltf and Shprh proteins are E3 ligases which polyubiquitinate PCNA. They were also reported to be involved in the regulation of monoubiquitination of TLS under very high UV doses [44]. The normal TLS observed in the *Shprh^−/−^Hltf^−/−^* cells suggests that PCNA-mUb rather than PCNA-polyUb is involved. However, it was recently reported that PCNA polyubiquitination is reduced, but not completely eliminated in *Shprh^−/−^Hltf^−/−^* MEFs, suggesting that an additional E3 ligase acts on PCNA [38]. Thus, an involvement of PCNA-polyUb in TLS cannot be ruled out based on these experiments alone. However, given the normal TLS in *Shprh^−/−^Hltf^−/−^* MEFs, and the biochemical data on the binding of TLS polymerases to PCNA-mUb [13], [22], [23], [45], it does seem that the dependence on ubiquitination is primarily due to the activity of PCNA-mUb.

The chromosomal significance of these finding is indicated by the faster accumulation of Rpa foci in *Pcna^K164R/K164R^* cells UV irradiated at the G1/S boundary of the cell cycle, and their slower clearance compared to *Pcna^+/+^* cells. Rpa strongly binds sites of ssDNA, providing a convenient tool for assaying ssDNA gaps caused by UV lesions during replication. Recent data suggest that at least in MEFs, TLS is the major pathway for repair of replication gaps caused by UV lesions [31]. Moreover, we have recently found that the disappearance of UV-induced Rpa foci is strongly reduced in cells in which the expression of polζ was knocked down, indicating involvement of TLS [33]. Thus, the inhibition of the clearance of post-UV Rpa foci in *Pcna^K164R/K164R^* cells is consistent with the decreased TLS across the TT CPD and TT 6-4 PP lesions observed in the gapped plasmid system, providing further support to the importance of PCNA-Ub for maximal TLS.

The debate on the role of PCNA-Ub in mammalian TLS involved primarily the activity of polη in bypassing UV lesions, where a series of papers presented conflicting results [13], [22]–[24], [27]. Those studies were based on mutating, or even entirely deleting, the polη ubiquitin-binding domain. Our study directly addressed ubiquitinated PCNA, and using functional TLS assays showed that TLS across TT CPD was impaired in the absence of PCNA ubiquitination, indicating that PCNA-Ub is required for the maximal bypass activity of polη. Two studies reported that PCNA-mUb was not required for polη-promoted TLS across TT CPD in a cell-free TLS assay [46], [47] (but was required to bypass an N-2-acetylaminofluorene-guanine adduct; [47]). These cell-free systems may not have faithfully mimicked the *in vivo* requirements for polη-promoted TLS across TT CPD due to the inherent ability of purified polη to bypass a TT CPD unassisted by any other protein [9], [10]. Adding our new data to the previously published data, we conclude that in a sense both sides of the controversy on the role of PCNA-Ub in TLS were right: On one hand we clearly show that TLS across three different DNA lesions, TT CPD, TT 6-4 PP and cisPt-GG, requires PCNA-Ub for maximal activity, but on the other hand TLS across each of the three lesions occurs also in the absence of PCNA-Ub, albeit at reduced extent and altered mutagenic specificity.

A key issue in TLS is the mechanism that ensures the recruitment of TLS polymerases to their cognate lesions, such that the entire TLS system operates without causing excessive mutations. This mechanism is regulated by the tumor suppressor p53, exerting its effect, at least in part, via the PCNA-binding function of the p21 protein, whose expression it regulates [2], [15]. Is PCNA-Ub an important regulator of this process of TLS fidelity control? TLS in this study was analyzed with lesions that span a broad range of bypass fidelity; From highly accurate TLS (TT CPD, <1% errors), via mostly accurate TLS (cisPt-GG, about 10% errors), up to mostly mutagenic TLS (TT 6-4 PP, about 65% errors). Some variations in mutagenic spectra among the wild-type MEFs were likely caused by differences in the genetic background of the MEFs, and by changes that might have occurred during immortalization. The absence of PCNA-Ub changed the fidelity of TLS across both the ‘accurate’ cisPt-GG lesion, as well as the mutagenic TT 6-4 PP lesion, but in different directions. Thus, while TLS across cisPt-GG became more mutagenic in the absence of PCNA-Ub (Figure 3D and Table S2), it became more accurate for the bypass of TT 6-4 PP (Figure 3C and Table S2). It is easy to envisage that conditions that decrease the efficiency of TLS will also cause lower fidelity, like in the case of cisPt-GG, because the TLS machine operates under sub-optimal conditions. However, the observation that the absence of PCNA-Ub the lower TLS across TT 6-4 PP was associated with a higher accuracy is somewhat surprising. It suggests that maximal TLS for some lesions cannot be achieved without compromising fidelity. Thus, higher TLS does not necessarily mean higher fidelity, and for some lesions the advantage of more efficient TLS outweighs the cost of decreased fidelity. In the case of TT CPD TLS was very accurate in both cells types, arguing that the lack of PCNA-Ub did not cause a major change in the fidelity of TLS across this type of lesion. In summary, PCNA-Ub affects not only the efficiency of TLS, but also its mutagenicity.

Our data show that a significant fraction of TLS in mammalian cells occurs in the absence of PCNA ubiquitination. This situation is different from the TLS in *S. cerevisiae*, where PCNA ubiquitination is essential for TLS [12], [48]. The situation in chicken DT40 cells is more complex. It was proposed that Rev1 and PCNA-Ub function in distinct mechanisms that control TLS, and that PCNA-Ub is essential for filling postreplication gaps but not for fork progression, whereas Rev1-dependent TLS is important at stalled forks, but does not play a central role in gap filling [19]. Analysis of TLS across a site-specific TT 6-4 PP adduct in a plasmid showed normal activity in *PcnaK164R* mutant DT40 cells [20]. Thus, in DT40 cells, there is evidence for PCNA-Ub independent TLS. The situation in mammalian cells appears to be different, with a less distinct division of function between PCNA-Ub and Rev1 at postreplication gaps and stalled forks, respectively. Thus, unlike in DT40 cells, in mammalian cells both Rev1-dependent TLS [31], and PCNA-Ub (as described above) are important for filling in of postreplication gaps. The fact that each of polη, polζ, and Rev1 contribute to UV survival of cells carrying the PcnaK164R mutation, as shown above, provides strong evidence for the participation of these polymerases in PCNA-Ub independent TLS reactions. This is in contrast to a previous study with XPV human cells, in which the expression of PCNA was reduced using siRNA, and supplementing the PCNAK164R mutant from a plasmid did not increase UV sensitivity [49]. The lack of effect in that study might have been caused by background levels of endogenous PCNA.

**Figure 8:**
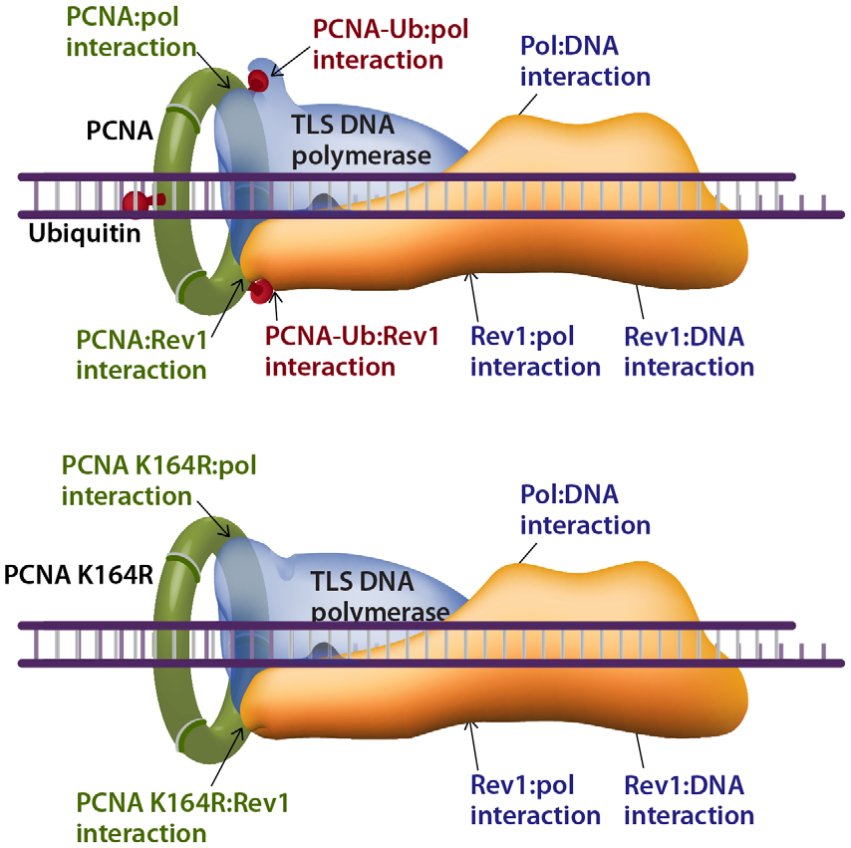
Model describing interactions that stabilize a TLS DNA polymerase during lesion bypass. The binding of a TLS DNA polymerase to a primer-template-lesion involves at least 7 known stabilizing interactions, 4 of which involve PCNA (top drawing). Two of these interactions, which involve the ubiquitin moiety, are lost in cells carrying the *PcnaK164R* mutation (lower drawing). The remaining 5 interactions (lower drawing) are sufficient to promote TLS, albeit with lower efficiency and altered mutagenic specificity. See text for details.

How does TLS operate in the absence of PCNA-Ub? A possible explanation can be proposed by considering the interactions that stabilize the TLS machinery acting on a damaged template. The composition of the TLS machinery is not fully understood, and neither is the composition of TLS complexes. However, based on the current knowledge we propose a model that includes a TLS complex with a minimal number of 3 proteins, namely the TLS DNA polymerase, PCNA and the Rev1 protein, acting as a scaffold (Figure 8). Depending on the type of DNA damage, other proteins are likely to be involved. Such a complex involves 7 known stabilizing interactions (reviewed in [50]), which include the interactions of: (1) The TLS polymerase with the DNA. (2) The TLS polymerase (via the PIP domain) with PCNA. (3) The TLS polymerase with the ubiquitin at position K164 in PCNA. (4) The TLS polymerase with Rev1. (5) Rev1 with ssDNA. (6) Rev1 with PCNA. (7) Rev1 with the ubiquitin at position K164 in another monomer of PCNA (Figure 8). In cells with the *PcnaK164R* mutation, two of these interactions are lost – of the ubiquitin with the TLS polymerase and with Rev1 (Figure 8 lower panel). However, 5 stabilizing interactions are left, of which 3 directly involve the TLS polymerase: with PCNA, with the DNA, and with Rev1. Thus, TLS DNA polymerases are recruited to damaged sites in DNA also in the absence of PCNA ubiquitination, and the TLS machinery is stable enough to perform lesion bypass, although at reduced efficiency.

In conclusion, ubiquitinated PCNA is required for maximal TLS across a variety of lesions, supporting the model of recruitment of TLS polymerases to the damaged DNA via interaction of their ubiquitin-binding domain to PCNA-mUb [22]. Yet, TLS polymerases can be recruited to damaged DNA in the absence of PCNA-Ub and perform TLS, and although secondary in efficiency under normal conditions, they do contribute to the protection of cells against DNA damage.

## Materials and Methods

### Cell cultures

The immortalized MEFs used in this study were prepared from mice with the following genotypes: *Pcna^+/+^* and *Pcna^K164R/K164R^* [29]; *Rad18^+/+^* and *Rad18^−/−^* [37]; *Hltf^+/+^Shprh^+/+^* and *Hltf^−/−^Shprh^−/−^* [38]; *Usp1^+/+^*, *Usp1^−/−^*, *Usp1^−/−^* complemented with wild-type *Usp1*, and *Usp1^−/−^* complemented with the inactive *Usp1*C90S mutant [40]. The immortalized MEFs were cultured in DMEM supplemented with 2 mM L-glutamine, 100 units/ml of penicillin, 100 µg/ml of streptomycin, and 10% FBS. The primary MEFs were cultured in DMEM supplemented with 2 mM L-glutamine, 100 units/ml of penicillin, 100 µg/ml of streptomycin, non-essential amino acids (Biological industries), 2-Mercaptoethanol 50 µM, and 15% FBS. The immortalized MEFs were incubated at 37°C in a 5% CO_2_ atmosphere. The primary MEFs were incubated at 37°C in a 5% CO_2_ and 4% O_2_ atmosphere.

### Centrifugal elutriation

Separation of cells at G1/S phase of the cell cycle was performed by the elutriation method with the following modifications. The elutriation system consisted of a J6 Beckman elutriation centrifuge with a JE-5.0 rotor equipped with a single standard 5 ml elutriation chamber (Beckman Coulter, Inc., Fullerton, CA, USA), and a masterflex microprocessor pump drive, model 7524-05 (Cole Parmer). The elutriation medium was DMEM supplemented with 1% FBS, maintained at room temperature. The speed and temperature of the rotor were set constant at 3000 rpm and 25°C. Approximately 3×10^8^ *Pcna^+/+^* or *Pcna^K164R/K164R^* MEFs were harvested from cultures at ∼80% confluence, centrifuged, and suspended in 10 ml of DMEM (room temprature) supplemented with 1% FBS. Cell suspensions were introduced to the elutriation chamber at a flow rate of 50 ml/min. After 15 minutes the flow rate was increased by 10 ml/min and three 50 ml fractions were collected at this flow rate. The flow rate was then gradually increased to 160 ml/min in 10 ml/min increments. Three 50 ml fractions were collected after each subsequent increase of the flow rate. The G1/S fraction (analyzed by FACS) was taken for the UV-induced Rpa foci assay.

### Rpa foci assay

For Rpa immunostaining [33], *Pcna^+/+^* and *Pcna^K164R/K164R^* MEFs were fractionated by centrifugal elutriation, and cells in the G1/S boundary were seeded on 13 mm glass cover slips coated with 0.01% poly-L-lysine. After 2 h when the cells attached to the slides, the medium was removed and the cells were UV-C irradiated at 254 nm using a low-pressure mercury lamp (TUV 15w G15T8, Philips) at doses of 8 J/m^2^. The dose rate was measured using an UVX Radiometer (UVP) equipped with a 254-nm detector. At various time points after irradiation the cells were washed three times with PBS, pre-extracted with 25 mM HEPES pH 7.4, 50 mM NaCl, 3 mM MgCl_2_, 300 mM sucrose, 1% Triton X-100 for 5 minutes on ice with gentle shaking, and washed for three more times with PBS. The slides were then fixed in 4% paraformaldehyde for 15 minutes at room temperature and washed three times in PBS. Blocking was done in PBS supplemented with 5% normal goat serum for 30 minutes on ice. The cells were incubated for 4 hours on ice with anti-Rpa32 antibodies (AbCam, cat. No. ab2175) that were diluted 1∶200 in blocking solution. After incubation the slides were washed three times in PBS and incubated with a secondary antibody –goat anti mouse Alexa Fluor 488 (green) diluted 1∶1000, and with DAPI diluted 1∶1000 (both in blocking solution) for 45 minutes on ice. The slides were then washed three times in PBS and mounted on microscope slides using Aqua poly/Mount. Images were captured with a DeltaVision system (Applied Precision) equipped with an Olympus IX71 microscope. Optical images were acquired using CCD camera (Photometrics, Coolsnap HQ) and a 60×/1.42 objective (Olympus). For each cell line at each time point at least 100 cells were counted and the percentage of cells exhibiting Rpa foci was determined.

### TLS assay in cultured mammalian cells

(Figure S1) The assay was performed as previously described [34], and the gapped plasmids with site-specific lesions used in this assay were prepared as previously described as follows: TT CPD and TT 6-4 PP [35]; cisPt-GG [6]. Briefly, cells were co-transfected with a DNA mixture containing 100 ng of a gapped-lesion plasmid (GP-TT-CPD, or GP-TT-6-4 PP, or GP-cisPt-GG; kan^R^), 100 ng of a control gapped plasmid without a lesion (GP20; cm^R^), and 5 µg of the carrier plasmid pUC18, using jetPEI/DNA complexes for the immortalized MEFs or the Lipofectamine 2000 for the primary MEFs. After allowing time for gap filling and lesion bypass, the plasmids were extracted from the cells using alkaline lysis conditions, and used to transform an *E. coli RecA* reporter strain. The percentage of plasmid repair, of which most occurs by TLS, was calculated by dividing the number of transformants obtained from the gap-lesion plasmid (kan^R^ colonies) by the number of transformants obtained from the control gapped-plasmid (cm^R^ colonies). A small fraction of gap-lesion plasmids can be repaired by non-TLS events, which involve formation of a DSB followed by DSB repair. These are observed as plasmid isolates with large deletions or insertions. To obtain precise TLS extents, the plasmid repair extents were multiplied by the fraction of TLS events out of all plasmid repair events, based on the DNA sequence analysis of the plasmids. To determine the DNA sequence changes that have occurred during plasmid repair, sequence analysis was carried using the TempliPhi DNA Sequencing Template Amplification Kit and the BigDye Terminator v1.1 Cycle Sequencing Kit. Reactions were analyzed by capillary electrophoresis on an ABI 3130XL Genetic Analyzer from Applied Biosystems.

### UV sensitivity assay

Two methods were used: depletion of ATP as a measure for viability, and colony forming ability after UV irradiation. For the viability ATP assay *Pcna^+/+^* and *Pcna^K164R/K164R^* MEFs were seeded in 96-well plates. At 24 h after the seeding, cells were washed twice with Hanks' buffer, and irradiated in Hanks' buffer with UV-C at 254 nm using a low-pressure mercury lamp (TUV 15w G15T8, Philips). UV dose rate was measured using an UVX Radiometer (UVP) equipped with a 254-nm detector. After irradiation, Hanks' buffer was removed and the cells were incubated in a fresh growing medium for additional 48 h. Viability was determined using the CellTiter-Glo Luminescent Cell Viability Assay (Promega). Luminescence was measured using an Infinite® M200 Luminometer (Tecan). Throughout the entire experiment, none of the samples reached cell confluency.

For the colony forming ability assay *Pcna^+/+^*and *Pcna^K164R/K164R^* immortalized MEFs were transfected with siRNA against TLS polymerases as described below, and incubated for 48 h. Cells were then trypsinized, counted, and plated in 10-cm Petri dishes. After incubation of 12 h, cells were UV irradiated as described above, and incubated in fresh medium for 10–12 days. Colonies were fixed and stained with 1% methylene blue (Sigma). Colony forming ability was calculated by dividing the number of colonies in UV-irradiated plates by the number of colonies in unirradiated plates with pre-transfected with the same siRNA.

### Knocking down the expression of TLS DNA polymerase genes

The expression of specific DNA polymerase genes was knocked-down in *Pcna^+/+^* and *Pcna^K164R/K164R^* MEFs by transfection with 50 nM of siRNA pools specific for *PolH*, *Rev3L* or *Rev1*. The siRNAs used were from Dharmacon as follows: *mRev3L* SMARTpool (M-04219), *mPolH* ON-TARGETplus SMARTpool (LU-063800), *mRev1* SMARTpool (M-041898), siGENOME non-targeting siRNA #5 (D-001210), ON-TARGETplus nontargeting Pool (D-001810). Transfection was carried out using HiPerFect (Qiagen), according to the manufacturer recommendations. The effectiveness of knocking down the expression of TLS polymerases was measured by RT-PCR using **t**otal RNA that was extracted from the cells 48 h after transfection with siRNA, using the Perfect-Pure RNA cultured cells kit (5 PRIME). A hundred ng of total RNA was used for cDNA synthesis and RT-PCR by Maxime RT-PCR PreMix kit (iNtRON BIOTECHNOLOGY) according to the manufacturer recommendations. The following primers were used for the RT-PCRs: 5′-GTGGTACGAGTCTTCGG-3′ and 5′-TCTTGTGACTCGGGCTG-3′ for *mREV3L*, 5′-GAAGCCCGAGCATTTGGTG-3′ and 5′-GCCTCTCCTCAAGTTCCAG-3′ for *mPOLH*, 5′-AGAACGGAGAATGATGGC-3′ and 5′-GGCCCAGGATCCTCAGGTTTGCACACAGG-3′ for *mRev1*, 5′-ACCACAGTCCATGCCATCAC-3′ and 5′-TCCACCACCCTGTTGCTGTA-3′ for *mGAPDH*. The results of knocking-down the expression of *PolH*, *Rev3L* and *Rev1* are shown in Figure S2.

## Supporting Information

Figure S1 Outline of the quantitative assay for TLS in cultured mammalian cells. Mammalian cells are transfected with a gap-lesion plasmid (kan^R^) containing a site specific lesion (indicated by a star), along with a gapped plasmid (cm^R^) without a lesion, and a carrier plasmid (amp^R^; pUC18). Following an incubation period the plasmids are extracted, and used to transform *E. coli* cells, which are then plated in parallel on kan-LB and cm-LB plates. The ratio of kan^R^/cm^R^ transformants represents the extent of plasmid repair. Individual colonies are picked, and their plasmid contents analyzed for mutations in the DNA region corresponding to the original site of the gap.
(TIFF)

Figure S2 Knockdown of the expression of TLS polymerases. RT-PCR of mRNA from wild type and *Pcna* ^K164R/K164R^ MEFs pretreated with siRNA against mouse *Rev3L*, *PolH*, and *Rev1*. Non-targeting siRNA was used as control. For each analysis, the effects of that siRNA were examined on mouse *Gapdh* mRNA expression.
(TIFF)

Table S1 TLS across TT CPD, TT 6-4 PP, and cisPt-GG adduct in *Pcna^+/+^* and *Pcna^K164R/K164R^ MEFs*. *Pcna^+/+^* and *Pcna^K164R/K164R^* MEFs were each transfected with a mixture containing the indicated gap-lesion plasmid (kan^R^) along with the control plasmid GP20 (cm^R^). Following incubation to allow TLS, the DNA was extracted and used to transform an *E. coli* indicator strain. Plasmid survival levels were calculated by the ratio of kan^R^/cm^R^ colonies. TLS levels were calculated by subtracting the fraction of non-TLS events (large insertions and deletions) from the corresponding plasmid repair values. Relative TLS extents were given as percentage relative to TLS assayed with isogenic wild type MEFs. Actual colony counts are presented for a typical experiment. Each point represents the average TLS level of 3–6 experiments.
(DOC)

Table S2 DNA sequence analysis of bypass events across TT CPD, TT 6-4 PP, and cisPt-GG adduct in *Pcna^+/+^* and *Pcna^K164R/K164R^* MEFs. Plasmids were extracted from kan^R^ colonies obtained in the experiments described in Table S1, and subjected to DNA sequence analysis. The sequences opposite the site of the lesions are shown in the 5′ to 3′ direction. Accurate TLS is represented by the sequence 5′-CAAC-3′ opposite TT CPD and TT 6-4 PP or 5′-GCCT-3′ opposite cisPt-GG adduct. The underlined nucleotides are those located opposite the original lesions. Mutations are presented by bold type. Δ represents a single-nucleotide deletion. Mutagenic TLS was calculated as the percentage of non-AA sequences inserted opposite the TT CPD and TT 6-4 PP or non-CC sequences inserted opposite the cisPt-GG adduct or mutations at the nucleotides flanking the lesions out of all TLS events (which do not include large insertions or deletions). Non-TLS events include big deletions and insertion.
(DOC)

Table S3 TLS across TT CPD, TT 6-4 PP, and cisPt-GG adduct in *Rad18^+/+^* and *Rad18^−/−^* MEFs. *Rad18^+/+^* and *Rad18^−/−^* MEFs were each transfected with a mixture containing the indicated gap-lesion plasmid (kan^R^) along with the control plasmid GP20 (cm^R^). Following incubation to allow TLS, the DNA was extracted and used to transform an *E. coli* indicator strain. TLS extents were determined as described in the legend to Table S1.
(DOC)

Table S4 DNA sequence analysis of the bypass events across TT CPD, TT 6-4 PP, and cisPt-GG adduct in *Rad18^+/+^* and *Rad18^−/−^* MEFs. Plasmids were extracted from kan^R^ colonies obtained in the experiments described in Table S3, and subjected to DNA sequence analysis. The sequences opposite the site of the lesions are shown in the 5′ to 3′ direction. Accurate TLS is represented by the sequence 5′-CAAC-3′ opposite TT CPD and TT 6-4 PP or 5′-GCCT-3′ opposite cisPt-GG adduct. The underlined nucleotides are those located opposite the original lesions. Mutations are presented by bold type. Δ represents a single-nucleotide deletion. Mutagenic TLS was calculated as the percentage of non-AA sequences inserted opposite the TT CPD and TT 6-4 PP or non-CC sequences inserted opposite the cisPt-GG adduct or mutations at the nucleotides flanking the lesions out of all TLS events (which do not include large insertions or deletions). Non-TLS events include big deletions and insertion.
(DOC)

Table S5 TLS across TT CPD, TT 6-4 PP, and cisPt-GG adduct in *Shprh^+/+^Hltf^+/+^* and *Shprh^−/−^Hltf^−/−^* MEFs. *Shprh^+/+^Hltf^+/+^* and *Shprh^−/−^Hltf^−/−^* MEFs were each transfected with a mixture containing the indicated gap-lesion plasmid (kan^R^) along with the control plasmid GP20 (cm^R^). Following incubation to allow TLS, the DNA was extracted and used to transform an *E. coli* indicator strain. TLS extents were determined as described in the legend to Table S1.
(DOC)

Table S6 DNA sequence analysis of bypass events across TT CPD, TT 6-4 PP, and cisPt-GG adduct in *Shprh^+/+^Hltf^+/+^* and *Shprh^−/−^Hltf^−/−^* MEFs. Plasmids were extracted from kan^R^ colonies obtained in the experiments described in Table S5, and subjected to DNA sequence analysis. The sequences opposite the site of the lesions are shown in the 5′ to 3′ direction. Accurate TLS is represented by the sequence 5′-CAAC-3′ opposite TT CPD and TT 6-4 PP or 5′-GCCT-3′ opposite cisPt-GG adduct. The underlined nucleotides are those located opposite the original lesions. Mutations are presented by bold type. Δ represents a single-nucleotide deletion. Mutagenic TLS was calculated as the percentage of non-AA sequences inserted opposite the TT CPD and TT 6-4 PP or non-CC sequences inserted opposite the cisPt-GG adduct or mutations at the nucleotides flanking the lesions out of all TLS events (which do not include large insertions or deletions). Non-TLS events include big deletions and insertion.
(DOC)

Table S7 TLS across TT CPD, TT 6-4 PP, and cisPt-GG adduct in *Usp1^+/+^*, *Usp1^−/−^*, *Usp1^−/−^* + WT Usp1 and *Usp1^−/−^* + Usp1 C90S MEFs. *Usp1^+/+^*, *Usp1^−/−^*, *Usp1^−/−^* + WT Usp1, and *Usp1^−/−^* + Usp1 C90S MEFs were each transfected with a mixture containing the indicated gap-lesion plasmid (kan^R^) along with the control plasmid GP20 (cm^R^). Following incubation to allow TLS, the DNA was extracted and used to transform an *E. coli* indicator strain. TLS extents were determined as described in the legend to Table S1.
(DOC)

Table S8 DNA sequence analysis of bypass events across TT CPD, TT 6-4 PP, and cisPt-GG adduct in *Usp1^+/+^*, *Usp1^−/−^*, *Usp1^−/−^* + WT Usp1 and *Usp1^−/−^* + Usp1 C90S MEFs. Plasmids were extracted from kan^R^ colonies obtained in the experiments described in Table S7, and subjected to DNA sequence analysis. The sequences opposite the site of the lesions are shown in the 5′ to 3′ direction. Accurate TLS is represented by the sequence 5′-CAAC-3′ opposite TT CPD and TT 6-4 PP or 5′-GCCT-3′ opposite cisPt-GG adduct. The underlined nucleotides are those located opposite the original lesions. Mutations are presented by bold type. Δ represents a single-nucleotide deletion. Mutagenic TLS was calculated as the percentage of non-AA sequences inserted opposite the TT CPD and TT 6-4 PP or non-CC sequences inserted opposite the cisPt-GG adduct or mutations at the nucleotides flanking the lesions out of all TLS events (which do not include large insertions or deletions). Non-TLS events include big deletions and insertion.
(DOC)
