# Supplementary figures and images for: PCNA Ubiquitination Is Important, But Not Essential for Translesion DNA Synthesis in Mammalian Cells
Source: PLoS Genet. 2011 Sep 8;7(9):e1002262. doi: 10.1371/journal.pgen.1002262 (PMC3169526; doi:10.1371/journal.pgen.1002262)

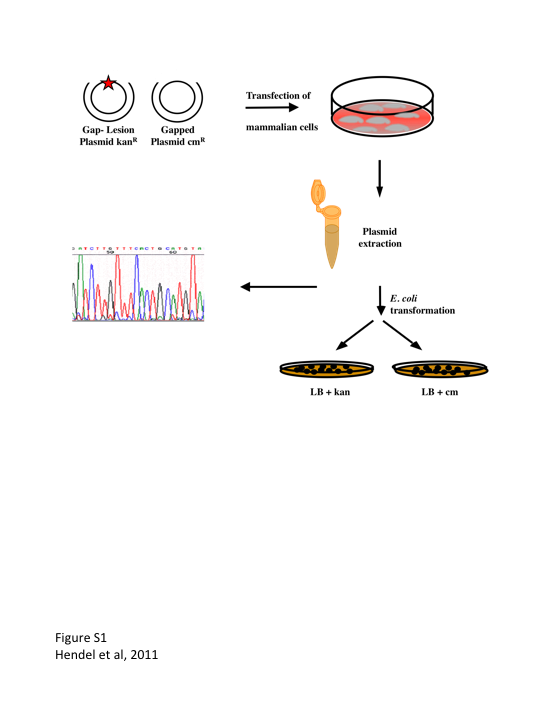

Supplement: Figure S1 — Outline of the quantitative assay for TLS in cultured mammalian cells. Mammalian cells are transfected with a gap-lesion plasmid (kanR) containing a site specific lesion (indicated by a star), along with a gapped plasmid (cmR) without a lesion, and a carrier plasmid (ampR; pUC18). Following an incubation period the plasmids are extracted, and used to transform E. coli cells, which are then plated in parallel on kan-LB and cm-LB plates. The ratio of kanR/cmR transformants represents the extent of plasmid repair. Individual colonies are picked, and their plasmid contents analyzed for mutations in the DNA region corresponding to the original site of the gap. (TIFF) [file pgen.1002262.s001.tiff]

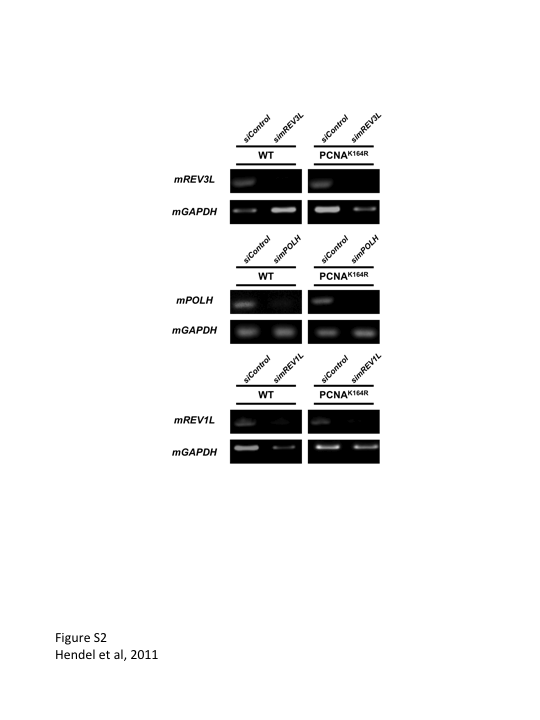

Supplement: Figure S2 — Knockdown of the expression of TLS polymerases. RT-PCR of mRNA from wild type and Pcna K164R/K164R MEFs pretreated with siRNA against mouse Rev3L, PolH, and Rev1. Non-targeting siRNA was used as control. For each analysis, the effects of that siRNA were examined on mouse Gapdh mRNA expression. (TIFF) [file pgen.1002262.s002.tiff]
