# Supplementary material for: PCNA Ubiquitination Is Important, But Not Essential for Translesion DNA Synthesis in Mammalian Cells
Source: PLoS Genet. 2011 Sep 8;7(9):e1002262. doi: 10.1371/journal.pgen.1002262 (PMC3169526; doi:10.1371/journal.pgen.1002262)
Supplement: Table S1 — TLS across TT CPD, TT 6-4 PP, and cisPt-GG adduct in Pcna+/+ and PcnaK164R/K164R MEFs. Pcna+/+ and PcnaK164R/K164R MEFs were each transfected with a mixture containing the indicated gap-lesion plasmid (kanR) along with the control plasmid GP20 (cmR). Following incubation to allow TLS, the DNA was extracted and used to transform an E. coli indicator strain. Plasmid survival levels were calculated by the ratio of kanR/cmR colonies. TLS levels were calculated by subtracting the fraction of non-TLS events (large insertions and deletions) from the corresponding plasmid repair values. Relative TLS extents were given as percentage relative to TLS assayed with isogenic wild type MEFs. Actual colony counts are presented for a typical experiment. Each point represents the average TLS level of 3–6 experiments. (DOC) [file pgen.1002262.s003.doc]

**Table S1. TLS across TT CPD, TT 6-4 PP, and cisPt-GG adduct in *Pcna^+/+^* and *Pcna^K164R/K164R^* MEFs**

| Cell line | Gap-lesion plasmid | Transformants | | Plasmid repair, % | TLS, % | Relative TLS, % |
| --- | --- | --- | --- | --- | --- | --- |
|  |  | KanR | CmR |  |  |  |
| *Pcna^+/+^* | TT CPD | 136 | 530 | 26±3 | 22±3 | 100±13 |
| *Pcna^K164R/K164R^* | TT CPD | 88 | 678 | 13±3 | 5±1 | 24±6 |
| *Pcna^+/+^* | TT 6-4 PP | 387 | 1281 | 36±3 | 33±3 | 100±8 |
| *Pcna^K164R/K164R^* | TT 6-4 PP | 160 | 978 | 16±1 | 10±1 | 31±3 |
| *Pcna^+/+^* | cisPt-GG | 245 | 1069 | 23±3 | 21±3 | 100±13 |
| *Pcna^K164R/K164R^* | cisPt-GG | 92 | 955 | 10±1 | 8±1 | 39±5 |
