# Supplementary material for: PCNA Ubiquitination Is Important, But Not Essential for Translesion DNA Synthesis in Mammalian Cells
Source: PLoS Genet. 2011 Sep 8;7(9):e1002262. doi: 10.1371/journal.pgen.1002262 (PMC3169526; doi:10.1371/journal.pgen.1002262)
Supplement: Table S5 — TLS across TT CPD, TT 6-4 PP, and cisPt-GG adduct in Shprh+/+Hltf+/+ and Shprh−/−Hltf−/− MEFs. Shprh+/+Hltf+/+ and Shprh−/−Hltf−/− MEFs were each transfected with a mixture containing the indicated gap-lesion plasmid (kanR) along with the control plasmid GP20 (cmR). Following incubation to allow TLS, the DNA was extracted and used to transform an E. coli indicator strain. TLS extents were determined as described in the legend to Table S1. (DOC) [file pgen.1002262.s007.doc]

**Table S5. TLS across TT CPD, TT 6-4 PP, and cisPt-GG adduct in *Shprh^+/+^Hltf^+/+^* and *Shprh^-/-^Hltf^-/-^* MEFs.**

| Cell line | Gap-lesion plasmid | Transformants | | Plasmid repair, % | TLS, % | Relative TLS, % |
| --- | --- | --- | --- | --- | --- | --- |
|  |  | KanR | CmR |  |  |  |
| *Hltf^+/+^* & *Shprh^+/+^* | TT CPD | 643 | 962 | 73±15 | 73±15 | 100±21 |
| *Hltf^-/-^* & *Shprh^-/-^* | TT CPD | 311 | 423 | 69±9 | 69±9 | 95±13 |
| *Hltf^+/+^* & *Shprh^+/+^* | TT 6-4 PP | 129 | 283 | 45±3 | 45±3 | 100±7 |
| *Hltf^-/-^* & *Shprh^-/-^* | TT 6-4 PP | 139 | 307 | 43±4 | 41±4 | 92±9 |
| *Hltf^+/+^* & *Shprh^+/+^* | cisPt-GG | 172 | 252 | 68±16 | 65±15 | 100±24 |
| *Hltf^-/-^* & *Shprh^-/-^* | cisPt-GG | 133 | 184 | 74±14 | 74±14 | 114±22 |
