# Supplementary material for: PCNA Ubiquitination Is Important, But Not Essential for Translesion DNA Synthesis in Mammalian Cells
Source: PLoS Genet. 2011 Sep 8;7(9):e1002262. doi: 10.1371/journal.pgen.1002262 (PMC3169526; doi:10.1371/journal.pgen.1002262)
Supplement: Table S8 — DNA sequence analysis of bypass events across TT CPD, TT 6-4 PP, and cisPt-GG adduct in Usp1+/+, Usp1−/−, Usp1−/− + WT Usp1 and Usp1−/− + Usp1 C90S MEFs. Plasmids were extracted from kanR colonies obtained in the experiments described in Table S7, and subjected to DNA sequence analysis. The sequences opposite the site of the lesions are shown in the 5′ to 3′ direction. Accurate TLS is represented by the sequence 5′-CAAC-3′ opposite TT CPD and TT 6-4 PP or 5′-GCCT-3′ opposite cisPt-GG adduct. The underlined nucleotides are those located opposite the original lesions. Mutations are presented by bold type. Δ represents a single-nucleotide deletion. Mutagenic TLS was calculated as the percentage of non-AA sequences inserted opposite the TT CPD and TT 6-4 PP or non-CC sequences inserted opposite the cisPt-GG adduct or mutations at the nucleotides flanking the lesions out of all TLS events (which do not include large insertions or deletions). Non-TLS events include big deletions and insertion. (DOC) [file pgen.1002262.s010.doc]

**Table S8. DNA sequence analysis of the bypass event type across TT CPD, TT 6-4 PP, and cisPt-GG adduct in *Usp1^+/+^*, *Usp1^-/-^*, *Usp1^-/-^* + WT Usp1 and *Usp1^-/-^* + Usp1 C90S MEFs.**

| TT CPD | | *Usp1^+/+^* | *Usp1^-/-^* | |
| --- | --- | --- | --- | --- |
| Nucleotide inserted opposite lesion | | Number of isolates (%) | | |
| C-AA-C | | 25 (52) | 33 (72) | |
| Non-TLS events | | 23 (48) | 13 (28) | |
| Total clones analyzed: | | 48 (100) | 46 (100) | |
| Accurate TLS frequency, % | | 100 | 100 | |
| Mutagenic TLS frequency, % | | <4 | <3 | |
| TT 6-4 PP | | *Usp1^+/+^* | *Usp1^-/-^* | |
| Nucleotide inserted opposite lesion | | Number of isolates (%) | | |
| C-AA-C | | 61 (64) | 48 (51) | |
| C-**C**A-C | | - | 1 (1) | |
| C-**T**A-C | | - | 1 (1) | |
| C-A**T**-C | | - | 1 (1) | |
| C-AA-**A** | | 5 (5) | 10 (11) | |
| **A**-AA-**A** | | - | 1 (1) | |
| C-**G**A-**A** | | 2 (2) | - | |
| C-A**Δ**-C | | 11 (12) | 8 (8) | |
| Non-TLS events | | 16 (17) | 24 (26) | |
| Total clones analyzed: | | 95 (100) | 94 (100) | |
| Accurate TLS frequency, % | | 77 | 68 | |
| Targeted mutagenic TLS frequency, % | | 14 | 16 | |
| Semi-targeted mutagenic TLS frequency, % | | 6 | 16 | |
| Mixed mutagenic TLS frequency, % | | 3 | <1.4 | |
| CisPt-GG | *Usp1^+/+^* | | | *Usp1^-/-^* |
| Nucleotide inserted opposite lesion | Number of isolates (%) | | | |
| G-CC-T | 31 (54) | | | 56 (70) |
| G-**A**C-T | 4 (7) | | | 10 (13) |
| G-**G**C-T | - | | | 1 (1) |
| G-**T**C-T | - | | | 1 (1) |
| G-CC-**A** | - | | | 1 (1) |
| G-C**A**-**A** | - | | | 1 (1) |
| G-**AG**-**A** | - | | | 1 (1) |
| Non-TLS events | 22 (39) | | | 9 (11) |
| Total clones analyzed | 57 (100) | | | 80 (100) |
| Accurate TLS frequency, % | 89 | | | 79 |
| Mutagenic TLS frequency, % | 11 | | | 21 |
| CisPt-GG | *Usp1^-/-^* + WT Usp1 | | | *Usp1^-/-^* + Usp1 C90S |
| Nucleotide inserted opposite lesion | Number of isolates (%) | | | |
| G-CC-T | 37 (77) | | | 31 (66) |
| G-**A**C-T | 4 (8) | | | 3 (6) |
| G-CC-**C** | - | | | 1 (2) |
| Non-TLS events | 7 (15) | | | 12 (26) |
| Total clones analyzed | 48 (100) | | | 47 (100) |
